# Supplementary material for: Ultrasound imaging identifies life history variation in resident Cutthroat Trout
Source: PLoS One. 2021 Feb 3;16(2):e0246365. doi: 10.1371/journal.pone.0246365 (PMC7857566; doi:10.1371/journal.pone.0246365)

**S2 Fig. Length-frequency distribution of fish in wild, resident populations surveyed in the seven to eight months prior to spawning.** Light grey bars represent the frequency of all fish sampled in a given size range; dark bars represent the frequency of fish identified as maturing females. Note that sampling in Gunderson Ditch targeted fish between 100 - 150mm total length. Sampling in other populations did not target fish of any particular size range.


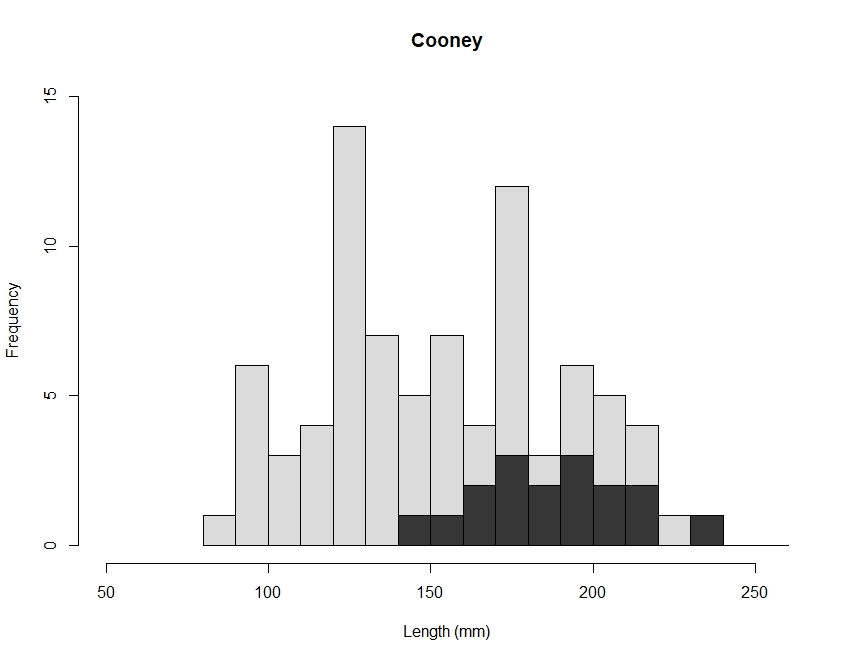

Supplement: S2 Fig — Light grey bars represent the frequency of all fish sampled in a given size range; dark bars represent the frequency of fish identified as maturing females. Note that sampling in Gunderson Ditch targeted fish between 100–150mm total length. Sampling in other populations did not target fish of any particular size range. (DOCX) [file pone.0246365.s002.docx]
