# Supplementary material for: Ultrasound imaging identifies life history variation in resident Cutthroat Trout
Source: PLoS One. 2021 Feb 3;16(2):e0246365. doi: 10.1371/journal.pone.0246365 (PMC7857566; doi:10.1371/journal.pone.0246365)

**S7 Fig. Length vs. age of fish captured in Gunderson Ditch.** Filled circles represent females with eggs; open circles reprent all other fish. Several age-1 fishe were larger than expected for this populations. Fish sampled from Gunderson Ditch likley may represent a mix of individuals spanwned and reared in upstream areas, as well as some spawned and reared within the ditch. Differences in rearing conditions (e.g., temperature, food avauilability, etc.) between the ditch and upstream areas could explain the broad variaion in size of in age-1 fish.


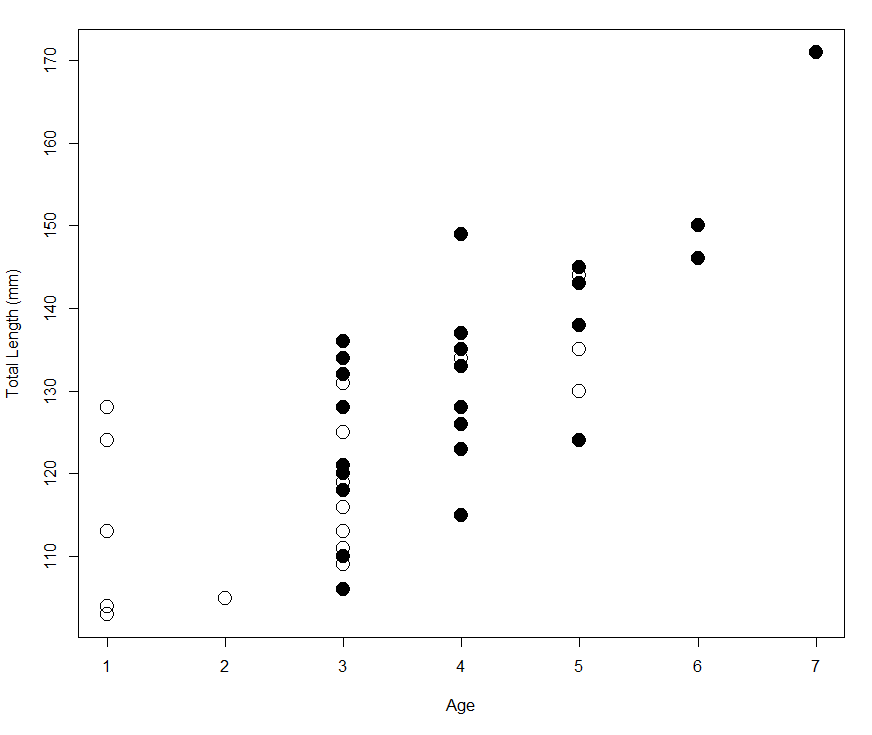

Supplement: S7 Fig — Filled circles represent females with eggs; open circles reprent all other fish. Several age-1 fishe were larger than expected for this populations. Fish sampled from Gunderson Ditch likley may represent a mix of individuals spanwned and reared in upstream areas, as well as some spawned and reared within the ditch. Differences in rearing conditions (e.g., temperature, food avauilability, etc.) between the ditch and upstream areas could explain the broad variaion in size of in age-1 fish. (DOCX) [file pone.0246365.s007.docx]
