# Supplementary material for: Ultrasound imaging identifies life history variation in resident Cutthroat Trout
Source: PLoS One. 2021 Feb 3;16(2):e0246365. doi: 10.1371/journal.pone.0246365 (PMC7857566; doi:10.1371/journal.pone.0246365)
Supplement: S1 Table — (DOCX) [file pone.0246365.s008.docx]

**S1 Table. Consistency in maturity classifications of individual fish at Sekokini Springs Hatchery over repeated examinations.**

| **Examination Dates** | **Sample Size** | **# Misclassifications** |
| --- | --- | --- |
| Repeated observations - 2018 Spawn Year |  |  |
| November and January | 5 | 0 |
| November and March | 17 | 0 |
| January and March | 30 | 0 |
| November, January and March | 3 | 0 |
| **Total** | **55** | **0** |
| Repeated observations - 2019 Spawn Year |  |  |
| October and January | 37 | 0 |
| October and March | 34 | 0 |
| January and March | 43 | 0 |
| October, January and March | 42 | 1 |
| **Total** | **156** | **1** |
